# Lateral flow urine lipoarabinomannan assay for tuberculosis diagnosis in HIV-positive inpatients and outpatients

**DOI:** 10.36416/1806-3756/e20250152

**Published:** 2025-09-22

**Authors:** Giovana Rodrigues Pereira, Márcia Silva Barbosa, Carina Secchi, Maiara Priscila dos Passos, André Kulzer Santos, Raimunda Sinthia Lima de Braga, Marina Scheffer de Souza, Gean Souza Ramos, João Vitor Vigne Duz, Denise Rossato Silva

**Affiliations:** 1. Programa de Pós-Graduação em Ciências Pneumológicas, Faculdade de Medicina, Universidade Federal do Rio Grande do Sul - UFRGS - Porto Alegre (RS) Brasil.; 2. Laboratório Municipal de Alvorada, Alvorada (RS) Brasil.; 3. Faculdade de Medicina, Universidade Federal do Rio Grande do Sul - UFRGS - Porto Alegre (RS) Brasil.; 4. Serviço de Pneumologia, Hospital de Clínicas de Porto Alegre - HCPA - Porto Alegre (RS) Brasil.

**Keywords:** Tuberculosis, Mycobacterium tuberculosis, HIV infections, Diagnosis

## Abstract

**Objective:**

To evaluate tuberculosis diagnosis with the use of the lateral flow urine lipoarabinomannan assay (LF-LAM) in addition to the Xpert^®^ MTB/RIF Ultra assay on sputum in HIV-positive inpatients and outpatients.

**Methods:**

This was a prospective cross-sectional study including HIV-positive patients ≥ 18 years of age with an indication for LF-LAM in accordance with Brazilian National Ministry of Health criteria.

**Results:**

A total of 140 patients were included in the study. LF-LAM was positive in 23 (16.4%). An additional 12 (8.6%) were diagnosed with the aid of LF-LAM, this increase in tuberculosis detection being statistically significant. LF-LAM-positive patients were younger and had lower CD4 counts in comparison with LF-LAM-negative patients. Smoking was more common among LF-LAM-negative patients than among LF-LAM-positive patients.

**Conclusions:**

The use of LF-LAM significantly increases the detection of tuberculosis in HIV-positive patients, mostly in those who are hospitalized. These findings highlight the utility of LF-LAM, especially in regions with high tuberculosis and HIV infection incidence.

## INTRODUCTION

The likelihood of developing active tuberculosis increases in proportion to the presence of certain risk factors or comorbidities such as HIV infection. According to the WHO, people living with HIV have an 18-fold higher risk of developing active tuberculosis than the rest of the world population. In 2019, of an estimated 815,000 HIV-associated tuberculosis cases worldwide, only 56% were reported, possibly in part due to underdiagnosis and suboptimal access to health care services. Therefore, improving access to early and rapid tuberculosis diagnosis is a fundamental principle of the fight against tuberculosis and a pillar of the WHO End TB strategy.^1^

Although molecular assays such as Xpert^®^ MTB/RIF (Cepheid, Sunnyvale, CA, USA) have helped in the diagnosis of tuberculosis by enabling faster and more sensitive results, especially in people living with HIV, there are still challenges in using such tests for tuberculosis diagnosis in some populations. In HIV-positive patients, the paucibacillary nature of tuberculosis, as well as scarce sputum production, makes it difficult to use molecular tests based on sputum samples.^1^

Lipoarabinomannan is an immunogenic virulence factor that is released by metabolically active or degrading bacterial cells and that is specific to mycobacterial species. Lipoarabinomannan was first characterized in 1980 as a potential marker of active tuberculosis and is the most studied biological marker for tuberculosis to date.^2^ Several studies have shown that, in tuberculosis patients, lipoarabinomannan is found in urine,^3^ ^,^ ^4^ as well as in blood and sputum.^5^ ^,^ ^6^ Urinary lipoarabinomannan levels are known to be elevated in individuals with tuberculosis-HIV coinfection and to increase with decreasing CD4 cell counts.^7^ The lateral flow urine lipoarabinomannan assay (LF-LAM) has been commercially available as a rapid point-of-care test that allows detection of mycobacterial lipoarabinomannan in urine samples.^8^ ^,^ ^9^ LF-LAM has a sensitivity of 42% for diagnosing tuberculosis in HIV-positive individuals with tuberculosis symptoms and of 35% for diagnosing tuberculosis in HIV-positive individuals who have not been evaluated for tuberculosis symptoms. As a simple point-of-care test that does not rely on sputum evaluation, LF-LAM can aid in the diagnosis of tuberculosis, particularly when a sputum sample cannot be produced.^10^

The objective of the present study was to evaluate tuberculosis diagnosis with the use of LF-LAM in addition to the Xpert^®^ MTB/RIF Ultra assay on sputum in HIV-positive inpatients and outpatients. 

## METHODS

### Study design and setting

A prospective cross-sectional study was conducted to evaluate LF-LAM for the diagnosis of tuberculosis in the routine care of HIV patients. The study was conducted in the city of Alvorada, in southern Brazil. 

### Patients and data collection

The study included HIV-positive inpatients and outpatients ≥ 18 years of age with an indication for LF-LAM in accordance with Brazilian National Ministry of Health criteria, as follows: having a CD4 count of < 100 cells/mm^3^, regardless of symptoms; showing signs and/or symptoms of pulmonary or extrapulmonary tuberculosis, regardless of CD4 count; and being severely ill, regardless of CD4 count. An adult was defined as being severely ill when presenting with any of the following: an RR ≥ 30 breaths/minute; an HR ≥ 120 bpm; inability to walk without assistance; and a body temperature ≥ 39°C, with local epidemiology and clinical judgment being taken into consideration. Patients who declined to participate in the study were excluded. 

Patients provided urine samples for LF-LAM, and the DETERMINE™ TB LAM Ag test (Abbott Laboratories, Abbott Park, IL, USA) was performed in accordance with the manufacturer instructions. The following data were recorded: demographic data (including sex, age, and race); symptoms (including cough, fever, night sweats, hemoptysis, weight loss, dyspnea, and chest pain); smoking status; alcohol use; CD4 cell count; sputum AFB smear and culture results; and Xpert^®^ MTB/RIF Ultra assay results. 

### Statistical analysis

Statistical analysis was performed with the IBM SPSS Statistics software package for Windows, version 22.0 (IBM Corporation, Armonk, NY, USA). Data were presented as number of cases, mean ± standard deviation, or median [interquartile range]. Categorical comparisons were performed with the chi-square test and Yates’ correction or Fisher’s exact test, as appropriate. Continuous variables were compared by means of the t-test or the Wilcoxon test. A two-sided value of p < 0.05 was considered significant for all analyses. 

## RESULTS

A total of 140 patients were included in the present study. The characteristics of the study population are shown in Table 1. The mean age was 30.3 ± 9.6 years, and 42.1% were male. Smoking, alcohol abuse, and drug use were reported by 71 (50.7%), 35 (25.0%), and 22 (15.7%), respectively. The mean CD4 count was 117.2 ± 25.9 cells/mm^3^. Thirty-seven (26.4%) were inpatients, and 103 (73.6%) were outpatients. 

**Table 1:** Characteristics of the study population.^a^

Characteristic	N = 140
Demographic characteristic	
Age, years	30.3 ± 9.6
Male	81 (42.1)
White	119 (85.0)
Symptom	
Cough	83 (59.3)
Weight loss	132 (94.3)
Dyspnea	10 (7.1)
Fever	11 (7.9)
Night sweats	13 (9.3)
Hemoptysis	1 (0.7)
Smoking	71 (50.7)
Alcohol abuse	35 (25.0)
Drug use	22 (15.7)
CD4 count	117.2 ± 25.9
Inpatient	37 (26.4)
Outpatient	103 (73.6)
Xpert^®^ MTB/RIF positivity (detected)	11 (7.9)
Rifampin resistance	2 (1.4)
LF-LAM	23 (16.4)

LF-LAM: lateral flow urine lipoarabinomannan assay. ^a^Data presented as mean ± SD or n/N (%).

The Xpert^®^ MTB/RIF Ultra assay on sputum was positive in 11 (7.9%) of the 140 patients included in the study, and resistance to rifampin was detected in 2 (1.4%). LF-LAM was positive in 23 (16.4%). An additional 12 patients (8.6%) were diagnosed with the aid of LF-LAM. This increase in tuberculosis detection was statistically significant (p = 0.0041). 

Table 2 shows the characteristics of patients with positive and negative LF-LAM results. LF-LAM-positive patients were younger than LF-LAM-negative patients (21.4 ± 3.8 years vs. 32.0 ± 9.5 years; p < 0.0001). Smoking was more common among LF-LAM-negative patients (n = 66; 56.4%; p = 0.005) than among LF-LAM-positive patients (n = 5; 21.7%). LF-LAM-positive patients had a lower CD4 count than did LF-LAM-negative patients (76.5 ± 8.1 cells/mm^3^ vs. 125.2 ± 20.1 cells/mm^3^; p < 0.0001). All of the patients with a positive LF-LAM result were inpatients, and 14 (12.0%) of those with a negative LF-LAM result were inpatients. 

**Table 2:** Comparison between patients with positive flow urine lipoarabinomannan assay results and those with negative results.^a^

Characteristic	LF-LAM-positive (n = 23)	LF-LAM-negative (n = 117)	p
Demographic characteristic			
Age, years	21.4 ± 3.8	32.0 ± 9.5	< 0.0001
Male	12 (52.2)	69 (59.0)	0.709
White	22 (95.7)	97 (82.9)	0.198
Symptom			
Cough	14 (60.9)	69 (59.0)	0.999
Weight loss	23 (100)	109 (93.2)	0.353
Dyspnea	0	10 (8.5)	0.368
Fever	1 (4.3)	10 (8.5)	0.692
Night sweats	1 (4.3)	12 (10.3)	0.694
Hemoptysis	0	1 (0.9)	0.999
Smoking	5 (21.7)	66 (56.4)	0.005
Alcohol abuse	4 (17.4)	31 (26.5)	0.510
Drug use	5 (21.7)	17 (14.5)	0.362
CD4 count	76.5 ± 8.1	125.2 ± 20.1	< 0.0001
Inpatient	23 (100)	14 (12.0)	< 0.0001
Xpert^®^ MTB/RIF positivity (detected)	3 (75.0)	8 (57.1)	0.999

LF-LAM: lateral flow urine lipoarabinomannan assay. ^a^Data presented as mean ± SD or n/N (%).

## DISCUSSION

In the present study, we found that LF-LAM was positive in 16.4% of HIV patients in a region of Brazil in which the prevalence of tuberculosis and HIV infection is high. An additional 12 patients (8.6%) were diagnosed with the aid of LF-LAM. All of the patients with a positive LF-LAM result were inpatients. LF-LAM-positive patients were younger and had a lower CD4 count than LF-LAM-negative patients. In addition, smoking was more common among LF-LAM-negative patients than among LF-LAM-positive patients. 

People living with HIV have an increased risk of tuberculosis, which accounts for approximately one third of all deaths in HIV patients.^1^ Therefore, among people living with HIV, systematic screening for tuberculosis disease should be conducted at each visit to a health care facility, according to the WHO.^11^ However, because there is often no sputum sample available for an Xpert MTB/RIF assay, LF-LAM has emerged as a diagnostic aid. Tuberculosis detection significantly increased from 11 (7.9%) with the use of the Xpert^®^ MTB/RIF Ultra assay alone to 23 (16.4%) when LF-LAM was added. This finding is in agreement with previous studies.^12^ ^,^ ^13^ In a prospective cohort of 98 HIV patients in Tanzania,^12^ the combination of the Xpert^®^ MTB/RIF assay and LF-LAM increased tuberculosis diagnosis from 24% to 37%. In a prospective cohort of 84 patients from three referral hospitals in Kilimanjaro,^13^ LF-LAM increased tuberculosis detection from 31% with the use of Xpert^®^ MTB/RIF alone to 55% with the use of both assays. In a systematic review and individual patient meta-analysis,^14^ the diagnostic yield of sputum Xpert in HIV-associated *Mycobacterium tuberculosis* bloodstream infection was 77% and increased to 89% when combined with LF-LAM. These findings are important because there is often no sputum sample available for testing with the Xpert^®^ MTB/RIF assay, especially in immunosuppressed patients. 

Although we evaluated outpatients and inpatients in our study, all positive results for LF-LAM were from hospitalized patients. Some studies^15^ ^,^ ^16^ have previously demonstrated that the use of LF-LAM may have a role in reducing mortality in hospitalized patients because of an increase in the number of tuberculosis diagnoses. In a pragmatic, randomized, parallel-group, multicenter trial^15^ conducted in ten hospitals in Africa, the authors evaluated the effect on mortality of a strategy including the use of LF-LAM. They found that this strategy was associated with reduced eight-week mortality. In another multicenter, double-blind, randomized controlled trial^16^ conducted in two hospitals in Malawi and South Africa, the use of LF-LAM in tuberculosis screening in HIV-positive inpatients did not reduce overall mortality in all patients, but it might have had benefits in some subgroups, such as those with low CD4 cell counts, those with severe anemia, or those with clinically suspected tuberculosis. Unfortunately, because we did not evaluate mortality in the present study, we were unable to evaluate the impact of those in-hospital diagnoses made with the aid of LF-LAM. 

In the present study, LF-LAM-positive patients had lower CD4 cell counts. In fact, it has been demonstrated that the estimated sensitivity of LF-LAM is greater in patients with lower CD4 cell counts.^17^ In a prospective, observational study including adult HIV patients hospitalized in a public district hospital in Malawi, LF-LAM was more likely to be positive in people with CD4 counts below 200 cell/mm^3^.^18^

An interesting finding of our study was that there was a lower frequency of smoking among patients with a positive LF-LAM result. Although it is possible that smoking can somehow hinder the detection of LAM, we found no other studies reporting a similar finding. Therefore, larger studies are needed to confirm this finding. 

Our study has limitations, given that we recruited patients from a single setting; however, we do not think that this prevents the generalization of the results. On the other hand, this is the first study in Brazil to evaluate the use of LF-LAM in outpatients and inpatients, in conjunction with the Xpert^®^ MTB/RIF Ultra assay on sputum. 

In conclusion, in the present pragmatic study we demonstrated that the use of LF-LAM significantly increases the detection of tuberculosis in HIV-positive patients, mostly in those who are hospitalized. These findings highlight the utility of LF-LAM, especially in regions with high tuberculosis and HIV infection incidence. Future multicenter studies involving a larger number of patients may help to confirm our findings. 
